# Integrated Semantics Service Platform for the Internet of Things: A Case Study of a Smart Office

**DOI:** 10.3390/s150102137

**Published:** 2015-01-19

**Authors:** Minwoo Ryu, Jaeho Kim, Jaeseok Yun

**Affiliations:** Embedded Software Convergence Research Center, Korea Electronics Technology Institute, 25 Saenari-ro, Bundang-gu, Seongnam 463070, Korea; E-Mails: minu@keti.re.kr (M.R.); jhkim@keti.re.kr (J.K.)

**Keywords:** Internet of Things, semantic interoperability, IoT service platform, semantic technology, top-level ontology

## Abstract

The Internet of Things (IoT) allows machines and devices in the world to connect with each other and generate a huge amount of data, which has a great potential to provide useful knowledge across service domains. Combining the context of IoT with semantic technologies, we can build integrated semantic systems to support semantic interoperability. In this paper, we propose an integrated semantic service platform (ISSP) to support ontological models in various IoT-based service domains of a smart city. In particular, we address three main problems for providing integrated semantic services together with IoT systems: semantic discovery, dynamic semantic representation, and semantic data repository for IoT resources. To show the feasibility of the ISSP, we develop a prototype service for a smart office using the ISSP, which can provide a preset, personalized office environment by interpreting user text input via a smartphone. We also discuss a scenario to show how the ISSP-based method would help build a smart city, where services in each service domain can discover and exploit IoT resources that are wanted across domains. We expect that our method could eventually contribute to providing people in a smart city with more integrated, comprehensive services based on semantic interoperability.

## 1. Introduction

The term Internet of Things (IoT), coined by Ashton in 1999 [1], has been a growing technological trend in recent years. IoT represents a technological revolution where the current Internet would be interconnected with physical objects and devices, so-called things, and their virtual representation due to several technological advances, including identification and contactless data exchange (RFID [2] and NFC [3]), distributed sensor network [4], short-range wireless communication (ZigBee [5] and Bluetooth [6]) and universal mobile accessibility (Wi-Fi hotspots [7] and cellular networks [8]). Accordingly, things in the world will be able to share the information about the status change in their environment and then become smart and reactive to external stimuli, eventually enabling one to create useful applications in various service domains [9–12].

**Figure 1.**
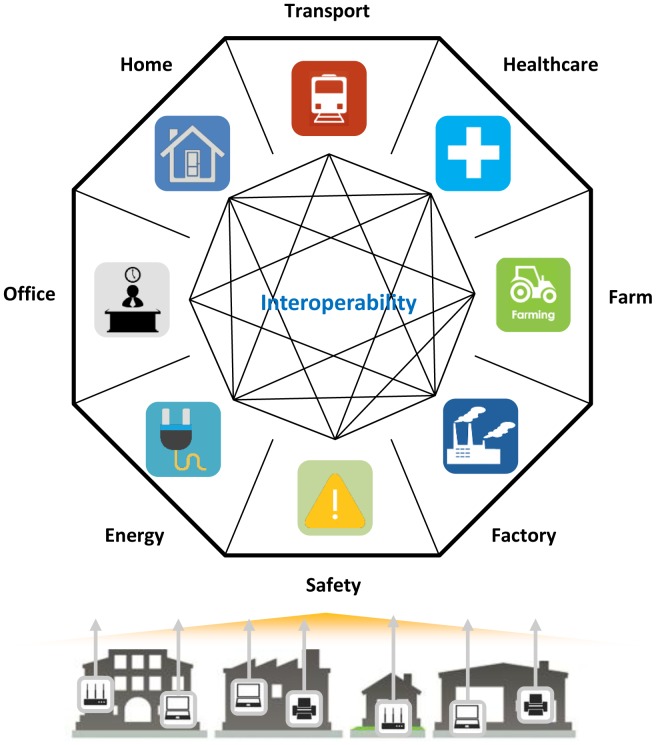
Interoperability between various service domains in a smart city.

In particular, IoT could be a key to unlocking the potential for providing integrated services across domains in a smart city. Due to existing vertical service architectures in a smart city, as in Figure 1, it has been difficult for services in each domain to discover and exploit resources in a city across service domains. IoT service architectures are thus evolving to horizontal service architectures from existing vertical service architectures in a smart city.

In horizontal service architectures, the goal of interoperability is to enable smart city services to share IoT resources (e.g., devices, virtual entity, data) between service domains and to better understand their surrounding environments, thereby providing end-users with intelligent services. For example, traffic information collected from a transportation domain can be used to adjust a wakeup call alarm service in a smart home with respect to a desirable subway departure time. Likewise, a combination of smart home and smart energy utilities can provide a new mashup service for saving energy according to usage patterns of home appliances. In other words, by applying horizontal architectures to a smart city, this will lead to the creation of new services and improve existing services via interoperability between service domains.

Accordingly, as Barnaghi illustrated with the context of IoT and semantics [13], the collected data within each service domain needs to be represented as semantic data, including their meanings (e.g., service domain knowledge), so that services from different domains could interpret and understand the data for interoperability (perform semantic interoperability).

A common way for semantic interoperability between different domains is to share their service domain knowledge using the well-known semantic technologies [14]. However, to share the service domain knowledge, a service domain needs to interpret and understand the service domain knowledge corresponding to an explicit specification generated from the other service domains. Consequently, a realistic way for sharing the service domain knowledge is to provide an integrated service system infrastructure, including platforms and applications to store and handle the whole knowledge of the service domain using semantic technologies.

For the integrated service system infrastructure, however, due to the characteristics of IoT, such as being highly-distributed, heterogeneous, and resource-constrained, we have to resolve three main problems for providing integrated semantic services via applying the semantic technologies to IoT: (1) integrated semantic discovery in distributed IoT domains; (2) dynamic semantic representation between a myriad of IoT resources in real time; (3) semantic data repository to archive a large amount of data collected from IoT devices.

To resolve these problems, we propose an integrated semantic service platform (ISSP). The proposed ISSP handles and stores various service domain knowledge in a smart city using ontologies and then provides semantic interoperability between different service domains based on the integrated knowledge. To this end, the ISSP is designed to support ontological models in a smart city using a top-level ontology, eventually providing integrated and improved smart city services for citizens.

The ontological model is modeled to be a formal, explicit representation of knowledge within a service domain using an ontology. Subsequently, we develop a web-based authoring tool to create an ontology for each service domain, an IoT-based service integration ontology (IIO) as a top-level ontology to store and handle the ontologies created by the web-based authoring tool and a semantic descriptor for semantic translation of IoT resources used in each service domain. We also develop semantic discovery-based IoT device monitoring and control functions by working with external IoT service platforms (*i.e.*, IoT data repositories), which may exist in each service domain.

Finally, to show the feasibility of the ISSP, we develop a prototype service for an office domain using the ISSP. The prototype service provides a preset, personalized office environment according to the interpretation of user text inputted via a smartphone. For the service, we design an ontological office model for the interpretation of the user input text and develop a web app that allows users to request a smart office service that they want. We also develop IoT device monitoring and control functions to perform the requested services using IoT resources in the office domain. In the prototype service, the monitoring and control functions are provided by working with Mobius, an IoT service platform, which we have previously implemented in another research project for managing and storing the data collected from various IoT devices [15].

The rest of this paper is organized as follows. The existing related work in this research field is introduced in Section 2. The detail description of the IIO is presented in Section 3. The ISSP architecture and implementation are introduced in Section 4. Section 5 presents a performance evaluation of ISSP through a case study. This section also introduces the prototype service for a smart office domain developed for the evaluation of the ISSP. We discuss practical issues to apply the semantics technologies in IoT service domains in Section 6. Finally, we conclude our remarks in Section 7.

## 2. Related Work

The term semantic, started by Berners-Lee in 1998, is a framework technology for automated processing for not just between machines, but also between humans and machines through interpretation of meanings to resources. Actually, the goal of the Semantic Web is to allow both humans and machines to understand through semantic interoperability based on well-defined meanings in the current web. Currently, the Semantic Web is standardized by the World Wide Web consortium (W3C) [16].

The Semantic Web is composed of explicit metadata, ontologies and logical reasoning. The explicit metadata is expressed by linguistic techniques, such as extensible markup language (XML), resource description framework (RDF) and web ontology language (OWL). Additionally, the ontologies express meanings of data and relationships using knowledge representation (KR) [17]. Finally, the logical reasoning is used to infer new information based on meanings of data and relationships in ontologies. The Semantic Web has been growing and applied in various research fields.

Skillen *et al.* proposed help-on-demand services in pervasive environments [18]. In this article, the ontological user models are created according to user behaviors and needs. Additionally, they implemented a prototype system for help-on-demand services with semantic rule-based reasoning, which they defined in the article. Accordingly, the system enables semantic discovery-based services using an ontology and semantic rule-based reasoning. Saha *et al.* proposed an ontology for home energy management [19]. The developed ontology is compatible with the suggested upper merged ontology (SUMO) [20]. Hence, the ontology enables knowledge sharing with other SUMO-compliant ontologies. The ontology is also used to provide intelligent advice on efficient energy consumption. However, these research works do not support semantic interoperability within different service domains. This is because the works focused on providing an intelligent service within each service domain, which does not consider semantic interoperability through sharing represented knowledge of their service domains.

Harshal *et al.* proposed a framework for linked open data (LOD) about sensor data collected from the physical environment [21]. They introduced a publishing method for LOD. In this framework, RDF and SPARQL are used for semantic discovery between the LOD clouds [22]. This framework enables interoperability between platforms using a sensor dataset description for LOD. Wang *et al.* proposed knowledge representation for integrated semantic services in IoT [23]. They developed a description ontology and an application for the integration of existing models, and they evaluated the performance of their proposed description ontology. However, these works did not consider dynamic, semantic translation of their resources in real time. In IoT service domains, devices and their resources are added dynamically to service domains in real time. Accordingly, to share service knowledge between different service domains, for example, LOD or description ontology should be updated considering the dynamically added devices and their resources.

Dou *et al.* proposed OntoMerge, a web-based translation system [24]. In OntoMerge, an ontological framework is proposed to enable translation between various vocabularies. In OntoMerge, they also use first-order logic to define relationships between individuals in different ontologies based on the ontological framework. However, to apply the system in IoT, it has to support dynamically-added individuals (*i.e.*, IoT devices) and service domain knowledge in real time.

DOLCE is a top-level ontology, which provided the whole ontology for the European research groups in the development of the WonderWeb [25]. DOLCE allows one to share information between added ontologies via captured ontological categories underlying natural languages and human common-sense. However, it did not consider explicit specification for service domain knowledge, and thus, we probably need to convert or redefine the explicit specification for the service domain knowledge to provide semantic interoperability.

Hunter proposed the ABC model, a core ontology model for semantic interoperability [26]. The model is an extensible ontology for integrated information from multiple genres of multimedia content within digital libraries and archives. Through this ontology, Hunter enables semantic interoperability from different domains. Compton *et al.* introduced a similar work [27]. They proposed the semantic sensor network ontology (SSNO) for semantic interoperability in wireless sensor networks (WSNs). They considered various use cases according to stimulus from WSNs and reflected the use cases in the SSNO, supporting semantic interoperability in various WSN-based service domains.

Recently, many researchers have been trying to share represented knowledge within each service domain, including the KR for semantic interoperability in IoT services [28–30]. However, sharing the knowledge between different service domains requires one to handle the whole knowledge representing the IoT-based service domains. In this perspective, the works of Hunter and Compton echo our motivation in this paper.

## 3. IoT-Based Service Integration Ontology

**Figure 2.**
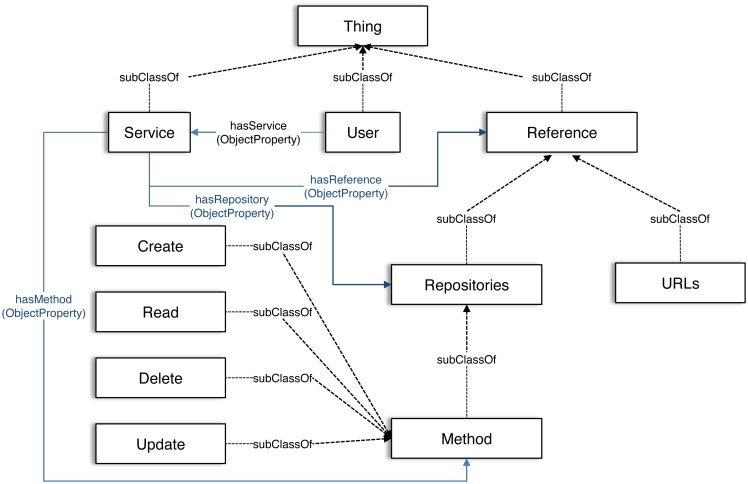
A schema of the IoT-based service integration ontology (IIO) to support ontologies created from various IoT-based service domains. The rectangle represents classes. The solid line represents a relationship between individuals, and the dashed line represents a relationship between super-class and subclasses.

In this section, we describe the IoT-based service integration ontology (IIO). The IIO is a top-level ontology to store and handle the ontologies added from each service domain to express their knowledge. We divide the entire knowledge of IoT-based services into three abstract concepts: service, user, and reference, as shown in Figure 2.

The class Service represents one or more IoT-based services in a smart city, and it has subclasses, each of which is a class Topic used to distinguish between the IoT services in the IIO. The class Topic is dynamically created and named by developers or administrators in each service domain using the web-based authoring tool, which we will explain in Section 4.1.1.

Individuals of the class Service have relationships with one or more individuals of the class Reference, Repositories, and Method through object properties asserted by hasReference, hasRepositories, and hasMethod, respectively.

The class User represents information about the end-users, such as the class of user name, class of user password, and class of user ID in the ontology. The classes for representing user information are created through the same web-based authoring tool as in the class Topic. Individuals of the class User have relationships with one or more individuals of the class Service through the object property expressed by the hasService.

The class Reference has two subclasses, URLs and Repositories, to represent the way to reference IoT resources located in external IoT service platforms. The class URLs represents uniform resource locators (URLs) of the external IoT service platforms. The class Repositories has a subclass Method that represents CRUD methods to reference IoT resources. As in User, the classes URLs and Method will have their individuals inputted from the authoring tool.

## 4. Integrated Semantic Service Platform

**Figure 3.**
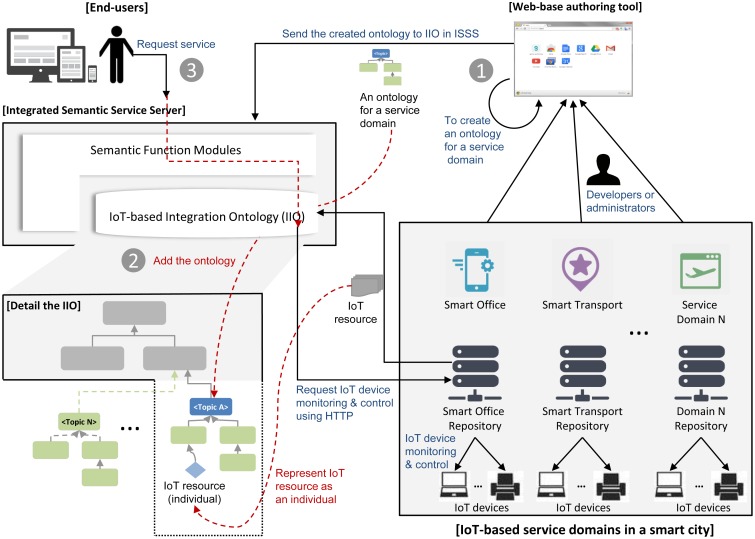
The overview of the integrated semantic service server (ISSS).

The ISSP provides an integrated semantic service through storing and handling the IIO and working with external IoT service platforms. The ISSP can also provide a web service via various devices, such as smartphones, tablets and personal computers. Figure 3 shows the overview of the ISSP.

In Figure 3, the ISSP is composed of the web-based authoring tool and integrated semantic service server (ISSS) to provide semantic interoperability between service domains in a smart city, as mentioned in Section 1.

First, developers or administrators in each service domain input values using the authoring tool to create an ontology based on their service domain knowledge, as shown in Figure 3-➀. At this time, the authoring tool creates an ontology corresponding to the values inputted and then sends the created ontology to the ISSS for adding it into the IIO, as shown in Figure 3-➁. In this procedure, the ISSS uses semantic functions to interpret the ontology and adds to it the IIO complying with the explicit specification of the IIO. This procedure also adds individuals of the class URLs and Method according to the input values for collaborating with external IoT service platforms (see the details in Section 4.1.1). Through these procedures, the ISSP is ready to provide interoperability between services in a smart city according to end-user requests. Finally, end-users can request for services (e.g., IoT device monitoring and control) with their applications, as shown in Figure 3-➂.

### 4.1. ISSP Architecture

**Figure 4.**
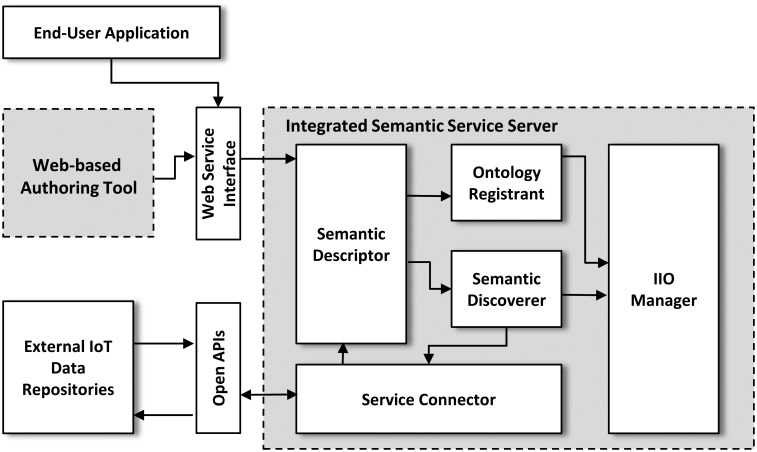
System architecture of the ISSP.

The ISSP consists of two main software packages: the web-based authoring tool and the integrated semantic service server (ISSS), as shown in Figure 4.

#### 4.1.1. Web-Based Authoring Tool

The web-based authoring tool is used to create an ontology using a web browser to add service domain knowledge within a smart city into the IIO without any development tool for the ontology. The web-based authoring tool provides four main input fields, as follows: (1) Service domain topic; (2) Ontology schema and relationship; (3) Reference resources; and (4) semantic web rule language (SWRL)-based rule.

The service domain topic field is used to allow developers or administrators in each service domain to create the class Topic. It will become a super-class of an ontology created with the values of the second input field to represent particular service domain knowledge in a smart city. This field also allows one to name the class Topic.

The ontology schema and relationship field is used to input the name of the class, object properties, data properties, domain, range, and restriction in order to create an ontology reflecting the service domain knowledge. Here, the web-based authoring tool does not support description logic (DL), such as its logic, SHOIN [31], when the object properties are inputted. Shallow or vocabulary-level ontologies (*i.e.*, these ontologies are made by RDF and OWL-lite) do not support the DL due to lower expression. Consequently, the web-based authoring tool uses SWRL [32] to support reasoning-based semantic services in the IIO.

The reference resource field is used to input information about IoT resources, such as URLs and CRUD methods (e.g., HTTP verbs or open APIs). At this time, the inputted values will become individuals of the class URLs and subclasses of the class Method corresponding to the value types, respectively.

The SWRL-based rule field is used to input SWRLs for reasoning based on added ontologies in the IIO.

#### 4.1.2. Integrated Semantic Service Server

The ISSS includes all of the semantic functions in the ISSP. The ISSS consists of five main components: the semantic descriptor, ontology registrant, semantic discoverer, service connector, and IIO manager. The semantic descriptor performs a semantic translation to represent values received from the web-based authoring tool and external IoT service platform as semantic data. The semantic descriptor has two semantic translations: schema-based semantic translation and individuals-based semantic translation. The schema-based semantic translation is used to represent values inputted from the authoring tool as OWL. In this translation, the semantic descriptor classifies the inputted values into classes, properties, and individuals, then converts them into an OWL based on RDF schema and OWL syntax endorsed by W3C, and finally sends it to the ontology registrant. The individual-based semantic translation is used to represent IoT resources received from an external IoT service platform as individuals of the IIO. Usually, an IoT service platform tends to have its own resource structure according to the standards (e.g., ETSI M2M or oneM2M) and service requirements in each service domain. Thus, the semantic descriptor needs to make RDFs based on the meanings of the resources defined in domain-specific forms. Finally, the semantic descriptor sends the RDFs to the ontology registrant.

The ontology registrant performs adding the OWL and RDFs received from the semantic descriptor into the IIO. The ontology registrant has two registration functions, including OWL registration and RDF registration. The OWL registration adds the ontology schema (*i.e.*, the received OWL) into the IIO to add individuals (*i.e.*, IoT resources) to the IIO. The OWL registration updates the IIO (*i.e.*, an OWL file) using the IIO manager according to its explicit specification. The RDF registration adds individuals (*i.e.*, the received RDFs) into the IIO according to the service added as an ontology schema to the IIO. To distinguish a service domain ontology among ontologies in the IIO, the RDF registration interprets explicit specification of the ontology schema in the IIO. The RDF registration next adds the individuals into the IIO in the same way as the OWL registration and repeats this until all of RDFs are added to the IIO. In the ontology registrant, the Pellet reasoner is used to interpret explicit specification of a particular ontology schema and the IIO so that the ontology registrant could appropriately add OWL and RDFs into the IIO.

The semantic discoverer performs semantic discoveries using SPARQL. SPARQL is used to make queries for semantic discoveries in the IIO. Through SPARQL, the semantic discoverer performs semantic discoveries over an ontology for a specific service domain, as well as two or more ontologies for various service domains in the IIO. The semantic discoverer can discover not only individuals related to requested services, but also their open APIs, which are individuals of the subclasses of the Method. To discover the individuals, the semantic discoverer uses the Pellet reasoner as the ontology registrant. The Pellet reasoner is used to interpret relationships between individuals and SWRLs defined through the fourth field (SWRL-based rule) in the web-based authoring tool. Finally, the semantic discoverer sends the related individuals and their open APIs to the service connector. This style of semantic discovery enables the ISSS to reach the IoT resources that end-user applications want, which are registered in external IoT service platforms with their IDs, URLs, and CRUD methods. Thus, the semantic discoverer would manage a sort of metadata about the IoT devices, whose real data and control values exist in external IoT repositories, *i.e.*, we perform discovery over their metadata of distributed storage.

The service connector generates commands for collaborating with external IoT platforms using the individuals received from the semantic discoverer. The service connector uses HTTP verbs according to the received open APIs. An individual of the class URLs is used as the network address to an external IoT service platform. The received individuals and open APIs are used as the arguments of query strings in HTTP to perform services requested from end-user applications. For example, assuming an individual of the class URLs is issp.com, the received individuals are Computer and off, each of which is the name of an IoT device and its operation mode, respectively, and if a related open API is PUT http:/<IoT service repository>/<Device Name>/<Device Value>, then the service connector generates command PUT http://issp.com/Computer?devicevalue=off. Through this procedure, the ISSP could provide semantic discovery-based IoT monitoring and control.

The IIO manager is responsible for handling and updating the IIO according to OWL and RDFs added from the ontology.

### 4.2. Implementation

**Figure 5.**
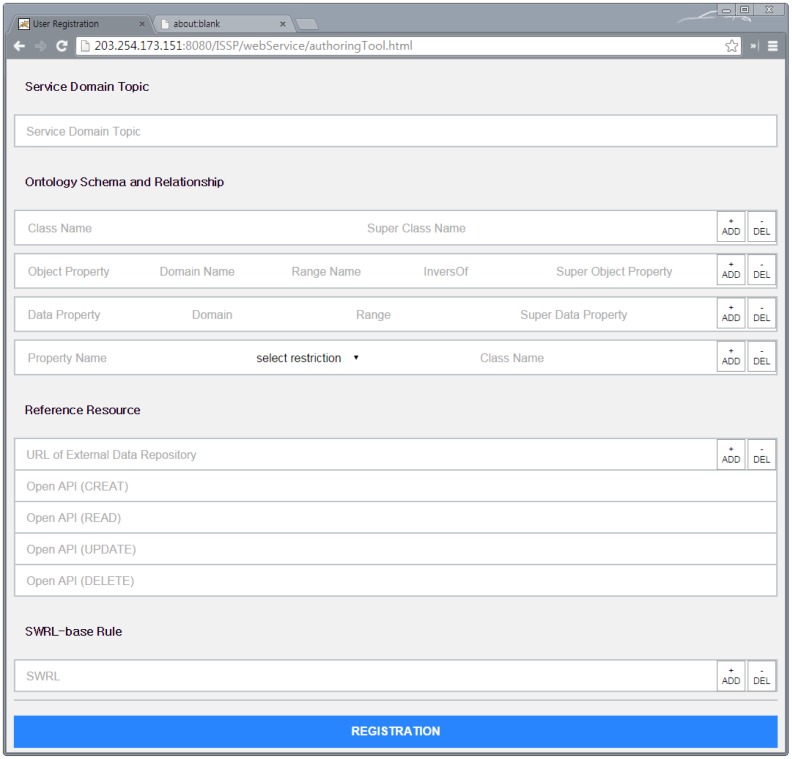
A snapshot of the web-based authoring tool.

In this section, we describe the implementation of the ISSP. Figure 5 shows the web-based authoring tool. The tool runs on a web browser, so as to support various devices, such as a tablet, smartphone and personal computer. We use the jQuery with the hypertext markup language (HTML). To support a large volume of ontologies, we can dynamically increase the number of each field by clicking the add button. In addition, we use the asynchronous JavaScript and XML (Ajax) to connect and send inputted values to the ISSS.

**Figure 6.**
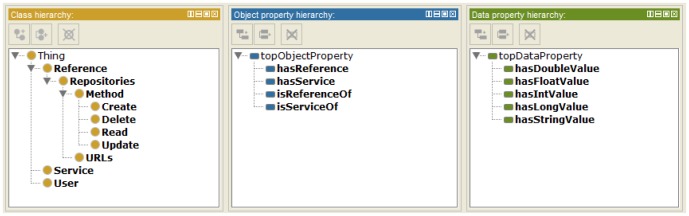
Hierarchy of classes, object properties, and data properties of the IIO: (from left), class hierarchy, object properties hierarchy, and data properties hierarchy

Figure 6 shows the hierarchy of classes, object properties, and data properties of the IIO using protégé 4.3 [33]. It allows specifying explicitly the ontology created from the web-based authoring tool. The IIO supports various primitive data types of IoT resources in each service domain using explicit data properties.

The ISSP is developed using Java, the Jena library, and the Pellet-2.3.1 reasoner on the web application server (WAS), *i.e.,* Tomcat 7.0. In the ISSP, we developed a java servlet for connection with the web-based authoring tool. We also developed the semantic descriptor, ontology registrant, and semantic discoverer using the Jena library and the Pellet-2.3.1 reasoner. In addition, we developed semantic query methods for the semantic discovery of individuals using SPAQRL in the IIO. Finally, the service connector is developed using the HTTP client library provided from Apache [34].

## 5. Performance Evaluation

In this section, we evaluate the performance of the ISSP by building a prototype service for a smart office: (1) adding an ontology created by the web-based authoring tool for the ontological office model into the ISSP; (2) dynamic semantic translation of the IoT resources using the semantic descriptor existing in the semantic service server in the ISSP; (3) performing IoT device monitoring and control using the dynamically-translated IoT resources and Mobius.

In (1) and (2), we use protégé for evaluating the ontology added and its individuals focused on schema and annotation according to the ontological office model. To evaluate (3), we show experimental results of device monitoring and control performed using a combination of the HTTP verbs for Mobius with its IoT resource represented as semantic data.

### 5.1. Case Study: Smart Office Application

**Figure 7.**
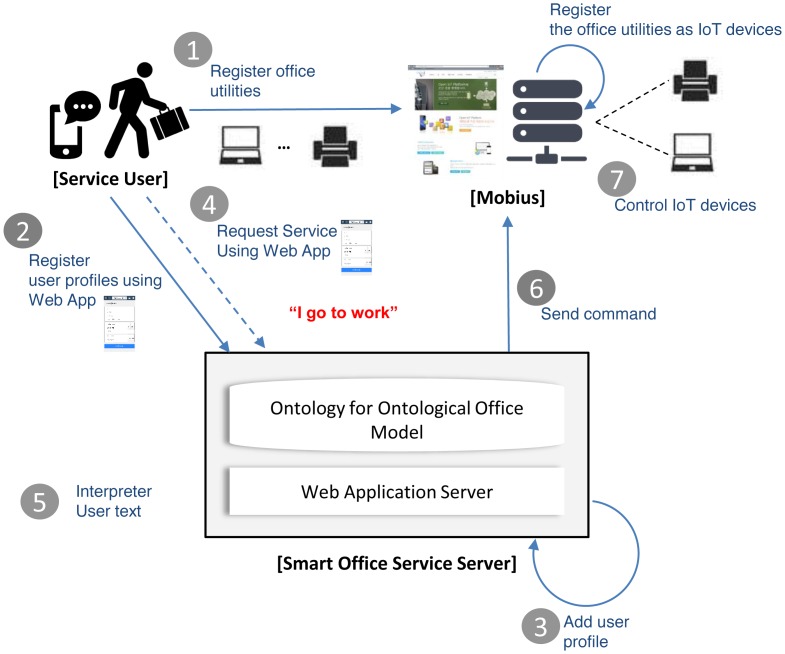
The service flow for the prototype service for the smart office.

A prototype smart office service is developed for the evaluation of the ISSP. The service provides a personalized office environment through semantic interpretations of inputted user text through the web app. To provide the service, it operates in two modes: (1) user profile registration mode; and (2) service request mode. The user profile registration mode is used to register user profiles, such as an ID, password, seat number, and user preset, for his or her personalized office environment, such as office actions and their corresponding devices and commands. The service request mode is used to request a smart office service based on the registered user profile and preset. The prototype service consists of the web app and ontological office model. Figure 7 shows a service flow for the prototype service.

In Figure 7, a user registers his/her office utilities into Mobius, as shown in Figure 7-➀. At this time, Mobius adds the office utilities to its repository. Then, the service user registers his/her profiles and commands the smart office service server to control the office utilities using the web app, as shown in Figure 7-➁. The registered user profile is used to create individuals according to explicit specification of the ontological office model. The smart office service server adds the registered user profile into the ontological office model, which is stored in a form of OWL file (*i.e.*, ontology), as shown in Figure 7-➂. Now, the service is ready to provide the smart office service according to user text input. In the service request mode, the service user can request a service using the web app, as shown in Figure 7-➃. In the procedure, a user text from the web app is interpreted according to the ontological office model in the smart office service server, and then, it sends the results of the interpretation to Mobius using open APIs provided by Mobius, as shown in Figure 7-➄,➅, respectively. Finally, Mobius performs an IoT device control corresponding to the received command, as shown in Figure 7-➆.

#### 5.1.1. Ontological Office Model

**Figure 8.**
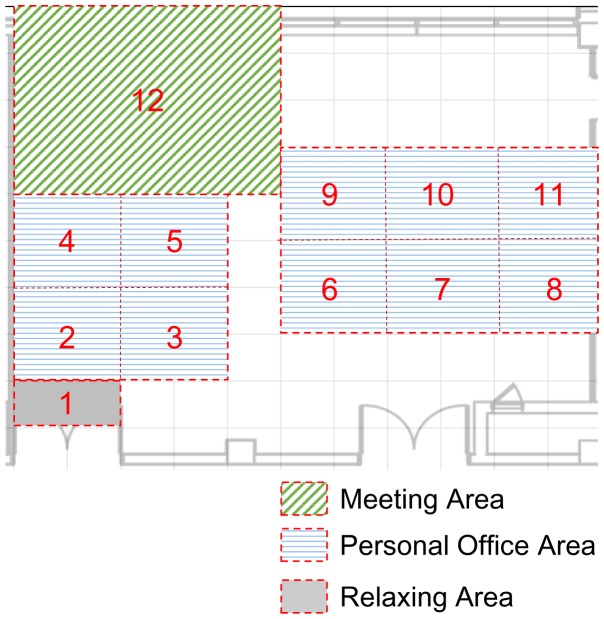
Classification of the space according to the characteristics.

The ontological office model is used to interpret the inputted user text and to control office utilities for the service. In the service, the ontological office model is designed based on an office located in our building (Korea Electronic Technology Institute). The office consists of personal office areas, a meeting area, and a relaxing area, as in Figure 8.

**Figure 9.**
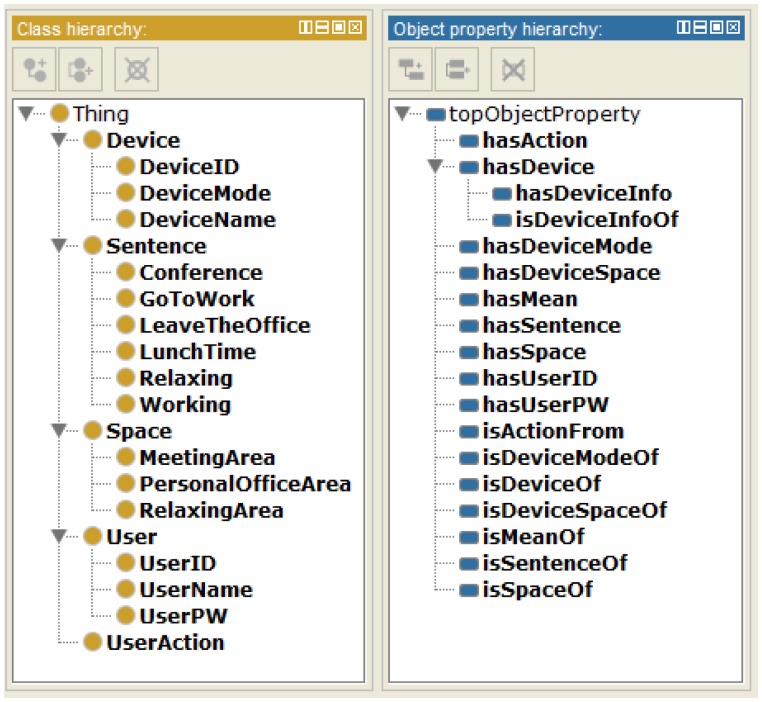
Hierarchy of classes and object properties.

Then, we chose six user office actions, such as get to work, meeting, leave the office, lunch time, relaxing, and working, through an interview with office workers. In the interview, we also surveyed relationships between user office actions and corresponding devices in each area. Finally, we developed the ontological office model using protege, as shown in Figure 9.

In Figure 9, the class Device represents an abstract class of office utilities corresponding user office actions. It has subclasses, such as the classes DeviceID, DeviceMode, and DeviceName, whose individuals express IDs, names, and operation modes of office utilities respectively. The individuals have relationships with one or more individuals of the classes User, UserAction and Space through the object property expressed by hasDeviceInfo, and hasSpace, respectively.

The class Sentence represents an abstract class of user texts inputted from the web app. It has subclasses, such as the classes GoToWork, Meeting, LeaveTheOffice, LunchTime, Relaxing, and Working. Individuals of the subclasses are added by the user profile registration mode via the web app. At the moment, the subclasses have the corresponding individuals according to the user action type inputted from the web app. The individuals also have relationships with one or more individuals of UserAction through the object property expressed by the hasAction.

The class User represents an abstract class of user information. It has subclasses UserID, UserName, and UserPW. Individuals of the subclasses are added the same way as the subclasses of the class Sentence. The individuals have relationships with one or more individuals of the classes Device, UserAction, and Sentence through object properties expressed by hasDeviceInfo, hasAction, hasSpace, and hasSentence, respectively.

The developed ontological office model is used to create and add an ontology in the evaluation of the IIO, *i.e.*, Evaluations (1) and (2).

#### 5.1.2. Mobius

Mobius is an oneM2M-compatible IoT service platform that we have previously implemented in another research project. It can manage and store the data collected from various IoT devices. Mobius provides capabilities for registration, management, and control of IoT devices. Mobius also provides open APIs to monitor and control IoT devices through RESTful interfaces.

### 5.2. Evaluation of the ISSP

**Figure 10.**
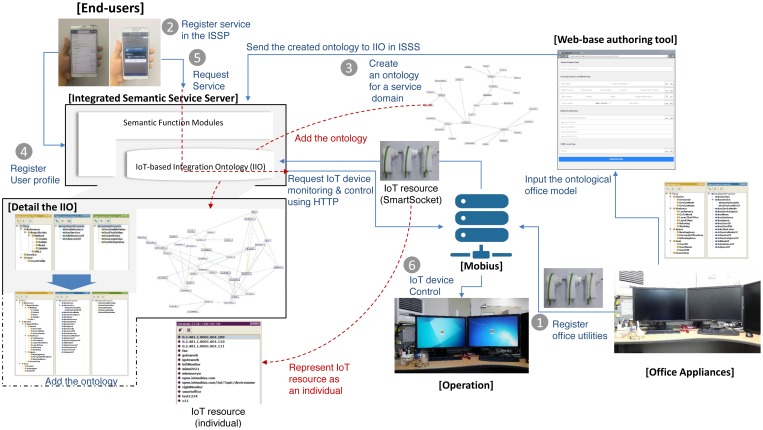
Evaluation process of the ISSP.

In this section, we describe the evaluation of the ISSP by applying the prototype service according to the following scenario: (1) register office utilities to Mobius; (2) register a service (*i.e.*, the web app) in the ISSP; (3) create an ontology based on the defined ontological office model using the web-based authoring tool and add the ontology into the IIO; (4) register user profile using the web app (registration mode); (5) request service using the web app (operation mode); and (6) verify the results of IoT device control, as shown in Figure 10.

**Figure 11.**
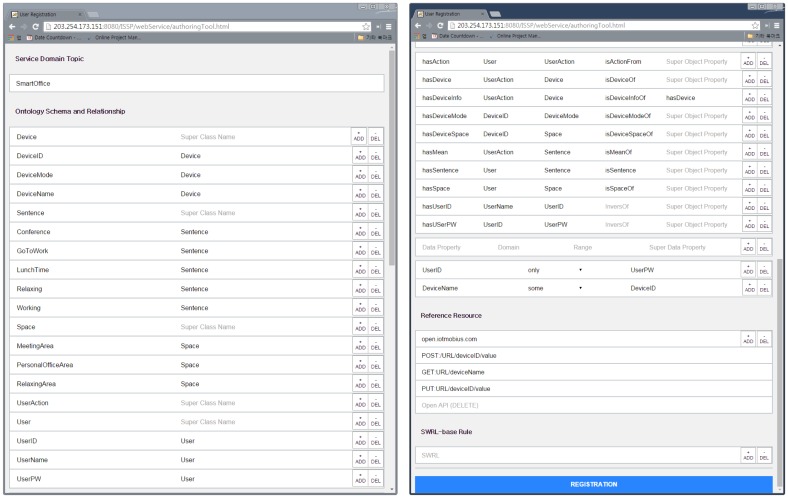
A snapshot of the inputted ontological office model using the web-based authoring tool.

**Figure 12.**
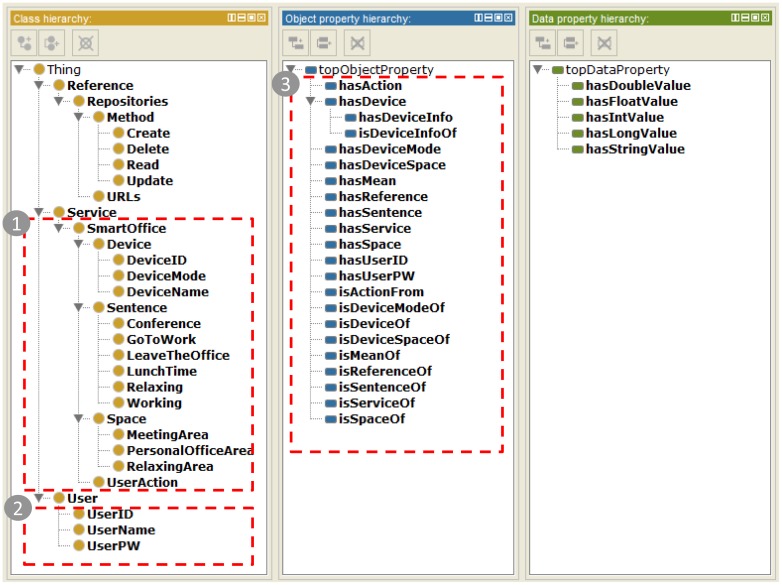
Result of the ontology added in the IIO with respect to the hierarchy of classes, object properties, and data properties.

**Figure 13.**
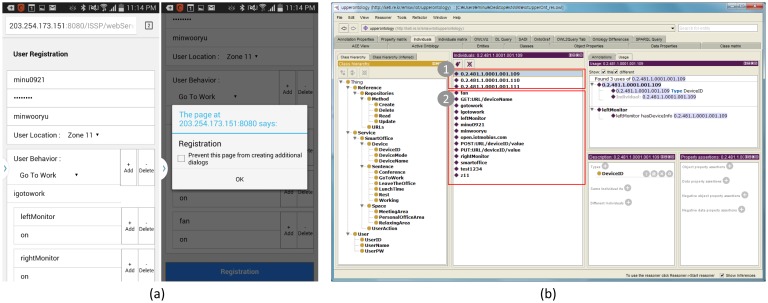
Results of (**a**) the web app operation (registration mode) and (**b**) individuals added in the IIO.

**Figure 14.**
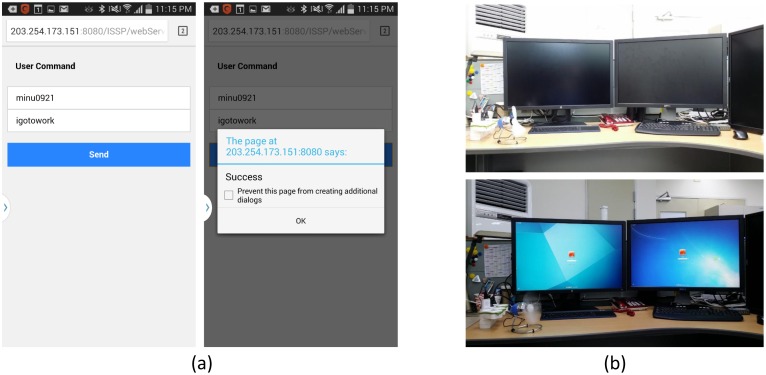
Results of (**a**) the web app operation (operation mode) and (**b**) IoT device control functions.

- Step 1: Register office utilities:
  We first perform registration of three office utilities, two monitors and a cool fan (named the left monitor, right monitor, and fan) to Mobius. To control each utility (*i.e.*, turn on or off), we use a smart socket consisting of a ZigBee-based radio transmitter and a relay [35]. During the registration, Mobius has obtained the unique IDs of the three devices, like “0.2.481. 1.0001.001.109”, “0.2.481.1.0001.001.110” and “0.2.481.1.0001.001.111”, respectively.
- Step 2: Register services:
  We register the web app for our smart office service. The web app is implemented for our smart office application using Ajax and operates in two modes, as mentioned in Section 5.1. To register the web app in the ISSP, we add two request parameters named “reqOfficeRegi” and “reqOperation” to the servlet in the ISSS. The ISSP is now ready to add user profile and preset to the IIO and to perform the smart office service according to user text input.
- Step 3: Create and add an ontology to the IIO:
  We create an ontology using the web-based authoring tool and add it to the IIO. We evaluate the first item (adding an ontology created by the web-based authoring tool for the ontological office model in the ISSP) using protégé. This step consists of creating an ontology through the ontological office model inputted using the web-based authoring tool and evaluation of the ontology added to the IIO using the ISSS.
  First, we input the ontological office model using the web-based authoring tool. The ontological office model is introduced in Section 5.1.1 in detail. To create an ontology of the ontological office model, we input the classes, properties, relationships, URL, and CRUD methods of Mobius, as shown in Figure 11. Here, we input SmartOffice as the name of the topic in the first field (service domain topic). We also input an ontology schema and relationship including classes, object properties, data properties, and relations between classes according to the explicit specification of the ontological office model in the second field (ontology schema and relationship). Next, we input the information of Mobius, including its URL (*i.e.*, open.iotmobius.com) and open APIs (GET:URL/deviceName>, POST:URL/deviceID/value and PUT:URL/deviceID/value) in the third field (reference resources). These will be used by the service connector for collaborating with the external IoT service platforms (*i.e.*, Mobius) according to the request of the end-user application. In this case study, we did not use the fourth field, SWRL.
  We also evaluate the ontology added to the IIO using the ISSS by analyzing the hierarchy of the ontology with protégé. To this end, we compare the hierarchy of the updated the IIO shown in Figure 12 with that of the original ontological office model shown in Figure 9).
  In Figure 12-➀, the class SmartOffice (the topic name of the service) is created as a subclass of the class Service in the IIO, and it has four subclasses asserted in the original ontological office model, except user classes, as explained in Section 3. The classes related to user information, including the classes UserPW, UserName, and UserID, are added as subclasses to User in the IIO, as shown in Figure 12-➁. Object properties of the office ontological model are added in the IIO, as shown in Figure 12-➂. We can thus know that the ontology is appropriately added in the IIO according to the explicit specification of the IIO, as well as the ontological office model.
- Step 4: Register user profile and preset using the web app:
  We evaluate the second item (dynamic semantic translation of the IoT resources using the semantic descriptor existing in the semantic service server in the ISSP) using protégé. Here, we investigate two types of individuals. The first type of individuals are represented with the user profile and preset inputted from the web app, whereas the second type of individuals is dynamically translated by working with Mobius. We register the user profiles and preset using the user profile registration mode in the web app as follows: (1) user ID: minu0921; (2) user PW: test1234; (3) user name: minwooryu; (4) user Location: zone11; (5) user behavior: GoToWork; (6) context: igotowork; and (7) device and use mode: ➀leftMonitor and on; ➁rightMonitor and on; ➂fan and on. Figure 13a shows the registration of the user profile and preset using the web app.
  In contrast, the second type of individuals is added in the IIO through periodic HTTP connections with Mobius (see the details in Section 4). At this time, the office utility names are used as arguments of the HTTP verbs for HTTP connection with Mobius, and then, Mobius returns the office utility IDs corresponding to the office utility names. This is a way to minimize the number of individuals in the IIO. As we explained in Section 4.1.2, an IoT device can be stored with different resource structures depending on the standard and the architecture of the IoT service platform, such as oneM2M or ETSI. For example, a single temperature sensor would be stored in an IoT service platform with related resources, including name, location, value, and the primitive data type of the value. In order to generate a formal, explicit representation of IoT resources located in various IoT service platforms, the ISSP needs to exploit all resources of the IoT devices and then represent them as individuals in real time, leading to building a huge, dynamic semantic data repository to store and handle all IoT resources in different IoT service platforms. Therefore, in our case, rather than constructing such a semantic data repository, we make use of the unique ID of IoT devices to distinguish, monitor, and control them.
  Figure 13b shows the result of the two types of individuals added in the IIO using protégé.
  In Figure 13b, the two rectangles classify two types of individuals. The first three individuals denote the office utility IDs added from Mobius by the HTTP connection, as shown in Figure 13b-➀. The rest of individuals denote the user profile, user preset and methods (*i.e.,* GET:URL/deviceName, POST:URL/deviceID/value, and PUT:URL/deviceID/value) inputted from the web app. From this result, we can know that the semantic descriptor appropriately represents the user profile and preset inputted from the web app as the first type of individuals. We also know that it appropriately represents office utility IDs inputted by working with Mobius as the second type of individuals.
- Step 5 and Step 6: Request service and IoT device control:
  In these steps, we evaluate the third item (performing IoT device monitoring and control using the dynamically-translated IoT resources and Mobius). We first input a user ID (*i.e.,* minu0921) and user command (*i.e.,* igotowork) registered from Step 4 and then send the inputted values to the ISSS using the service request mode of the web app, as shown in Figure 14a.
  We next evaluate the control functions of the office utilities, as shown in Figure 14b. From the experiment, we can know that the ISSP enables IoT device control through working with Mobius. In the control, the ISSP uses only office utility IDs and values for their operation modes. This means that the ISSP can perform IoT device monitoring and control using minimum semantic data by collaborating with our external IoT service platform, Mobius.

### 5.3. A Scenario of ISSP-Based Semantic Interoperability in a Smart City

**Figure 15.**
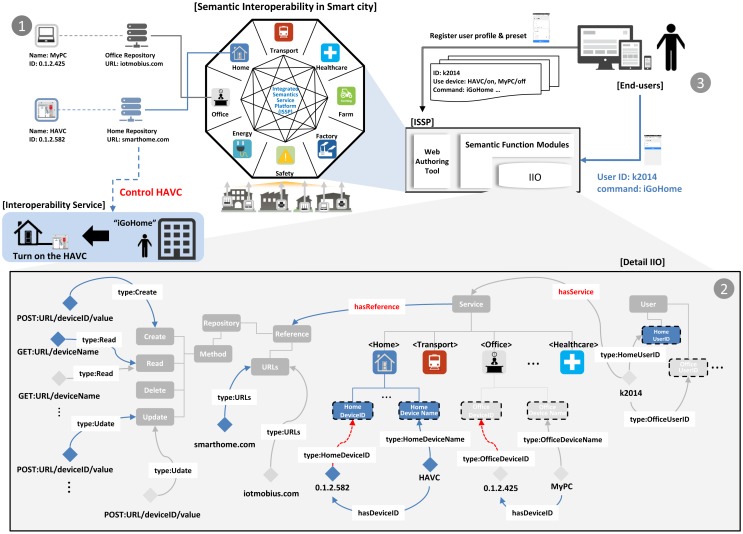
Semantic interoperability for a smart city service scenario.

In this section, we describe a scenario of ISSP-based semantic interoperability. In particular, we focus on how we can achieve interoperability between different service domains using the semantics in a smart city. We assume a scenario as follows: a service user wants to turn on the HVAC (heating, ventilation and air conditioning) system in his/her home when he/she finishes his/her office work. In other words, when a computer is turned off in the office, the user wants to turn on the HVAC. Figure 15 shows the semantic interoperability for the scenario.

A service user first registers his own devices (*i.e.,* HVAC and MyPC) at home and the office, to monitor and control them using the ISSP service, as shown in Figure 15-➀. Here, we assume that the user creates the same user ID between the home and office domains. Developers or administrators then add their service domain knowledge into the IIO using the tool, such as the rectangles with dashed lines in Figure 15-➁. After registration from service domains, the ISSP is ready to work with the service domains and discover related individuals in the IIO from the user.

The user registers his/her own profile and preset using a service application (e.g., mobile web), as in Figure 15-➂, where we assume that the profile and preset are as follows: (1) user ID: k2014; (2) user devices: HVAC and MyPC; (3) user device mode: HVAC → on. MyPC → off; and (4) command: iGoHome.

In this procedure, the ISSP defines relationships to provide interoperability via object properties specified from the IIO. For example, to define relationships between a service user and service domains, the ISSP discovers the matches with the inputted user ID (*i.e.,* k2014) in the class User and its subclasses, and then, the ISSP defines a relationship between the matched user ID and relevant service topics (*i.e.,* home and office) using an object property (*i.e.,* hasService). In addition, the ISSP can discover URLs *(i.e.*, iotmobius.com and smarthome.com) and CRUD methods (*i.e.*, GET:iotmobius.com/MyPC/, POST:iotmobius.com/MyPC/0.1.2.425, GET:smarthome.com/HVAC, POST:smarthome.com/HVAC/0.1.2.582) to work with external IoT service platforms, which are related to the available services of the user. Accordingly, the ISSP can monitor and control the devices having IDs corresponding to the device names inputted from the user.

The ISSP can now provide the user with the service according to the user command using semantic interoperability. The user sends his/her own ID and command (*i.e.*, “iGoHome”) to the ISSP, and then, the ISSP requests the office repository to turn MyPC off using the URL and CRUD methods in the office repository. After this procedure, the ISSP checks the use mode of MyPC. Once the use mode of MyPC goes off, the ISSP requests the home repository to turn the HVAC on in the same way as the MyPC is controlled. Therefore, the ISSP will be able to allow services to be provided across different service domains in a smart city based on semantic interoperability.

## 6. Discussion

As we explained regarding the importance of interoperability across the entire service domain (e.g., smart cities) in this paper, we need to provide an integrated service system infrastructure that can handle the whole service knowledge of IoT-based service domains to share IoT resources between service domains. For providing such integrated service system infrastructures, we have developed the ISSP; however, we need to consider additional practical issues.

From the perspective of IoT, the same type of devices can exist in various service domains at the same time, and each one may be defined and stored as a different device in the corresponding IoT service platform (e.g., IoT data repository) according to the service requirements. At this time, the device may have its corresponding roles required in a certain service domain. For example, we assume that two monitors having an identical model number exist in an office domain and home domain, respectively. Then, the monitors would be defined with a different name, type, function, and role according to the service requirement of each service domain. In view of each service domain, of course, it is natural for the device to have a different definition according to the service domain.

However, considering an integrated service system infrastructure for semantic interoperability, such a domain-dependent definition for the same type of devices results in increasingly growing semantic data (*i.e.*, individuals) and complexity of the relationship between them due to the duplicate existence of individuals of the same type of devices. An ideal way to resolve the problem is to identify and then redefine the profiles (e.g., name and function) of the same type of IoT devices in the integrated service system, when IoT devices are added from each service domain, though it is also a challenging task to deal with a large number of IoT devices that may have the same type.

Another issue is to accommodate various, continually increasing applications for IoT. In IoT-based service domains, a way for providing IoT device monitoring and control services is to use smartphone applications or web applications. Although we combine the ISSP with the web application using Ajax by manually adding these into the prototype service, this could not support connecting numerous applications for IoT device monitoring and control. Hence, we have to consider an efficient way for accommodating a gradually increasing number of applications.

Although we have proposed an integrated service platform to apply semantics technologies to the IoT-based service domains, a primary goal of the semantics technologies is to share represented knowledge within each service domain using the current web. Hence, we can consider generating open accessible data for semantic interoperability between the IoT-based service domains. Additionally, we would be able to use the well-known LOD for generating the data and sharing the LOD between the service domains. However, this method may not be appropriate for providing semantic interoperability for IoT-based services, because it should be dynamically generated and then published to support the various IoT devices added and their collected data in real time.

## 7. Conclusions

In this paper, we have presented an integrated semantic service platform (ISSP) to support ontological models in various IoT-based service domains. In order for the ontological models to be a formal, explicit representation of knowledge within a service domain, we have developed a web-based authoring tool with which we can create an ontology for a service domain. We have also developed an IoT-based service integration ontology (IIO) as a top-level ontology to maintain and handle the ontologies created by the web-based authoring tool. In addition, we have created a semantic descriptor for semantic translation of the IoT resources used in each service domain. Finally, we can perform semantic discovery-based IoT device monitoring control by working with our external IoT service platform, Mobius. With the proposed method, we have tried to address three challenging problems for applying semantic technologies to highly-distributed, heterogeneous and resource-constrained IoT-based systems: integrated semantic discovery in highly-distributed IoT domains, dynamic semantic expression between a large number of IoT resources in real time, and a semantic data repository to archive a huge amount of data collected from IoT devices. To show the practical feasibility of our proposed method, we have developed a prototype service for a smart office, which can provide a preset, personalized office environment, for example office utilities or lighting can be automatically turned on or off according to user text input sent from his or her smartphone. Through the evaluation, we have shown that the ISSP can allow the prototype service to appropriately perform semantic discovery, dynamic translation, and eventually IoT device control in real time working with our IoT service platform. We have also discussed a scenario for ISSP-based semantic interoperability to provide users with integrated and comprehensive services across service domains in a smart city. Accordingly, we can conclude with confidence that the ISSP-based method can eventually contribute to realizing semantic interoperability across the entire service domain based on IoT-based systems and semantic technologies.
